# An efficient non-parametric feature calibration method for few-shot plant disease classification

**DOI:** 10.3389/fpls.2025.1541982

**Published:** 2025-05-19

**Authors:** Jiqing Li, Zhendong Yin, Dasen Li, Hongjun Zhang, Mingdong Xu

**Affiliations:** School of Electronics and Information Engineering, Harbin Institute of Technology, Harbin, China

**Keywords:** deep learning, few-shot learning, plant disease classification, feature calibration, image classification

## Abstract

The temporal and spatial irregularity of plant diseases results in insufficient image data for certain diseases, challenging traditional deep learning methods that rely on large amounts of manually annotated data for training. Few-shot learning has emerged as an effective solution to this problem. This paper proposes a method based on the Feature Adaptation Score (FAS) metric, which calculates the FAS for each feature layer in the Swin-TransformerV2 structure. By leveraging the strict positive correlation between FAS scores and test accuracy, we can identify the Swin-Transformer V2-F6 network structure suitable for few-shot plant disease classification without training the network. Furthermore, based on this network structure, we designed the Plant Disease Feature Calibration (PDFC) algorithm, which uses extracted features from the PlantVillage dataset to calibrate features from other datasets. Experiments demonstrate that the combination of the Swin-Transformer V2F6 network structure and the PDFC algorithm significantly improves the accuracy of few-shot plant disease classification, surpassing existing state-of-the-art models. Our research provides an efficient and accurate solution for few-shot plant disease classification, offering significant practical value.

## 1 Introduction

UNDER the rapid development of hardware and information technology, deep learning has gradually become the mainstream method in the field of image classification. In conventional deep learning image classification processes, a large number of images are required to train the model, allowing it to learn and remember the common features of the same category in the image classification task. This enables the model to exhibit remarkable performance during the testing phase. With the advancement of IoT technology, an increasing number of terminal devices in various fields are beginning to acquire massive amounts of images, prompting attempts to use deep learning for specific image classification tasks, such as plant disease classification (Khan et al., 2022a; Khan et al., 2022b; Bashir et al., 2023; Vishnoi et al., 2023). In this field, numerous studies (Afifi et al., 2020; Argüeso et al., 2020; Figueroa-Mata and Mata-Montero, 2020; Karami et al., 2020; Egusquiza et al., 2022) have confirmed the feasibility of using deep learning techniques for plant disease classification. However, in practical applications, due to the irregularities in time and space of crop disease occurrences, edge devices often struggle to obtain sufficient data on rare plant diseases. Researchers define this challenge as a few-shot classification task in the field of plant disease. The biggest challenge of this task is how to effectively extract crucial classification information from small sample sets of disease data.

Recent work has primarily focused on improving and innovating models for few-shot tasks through three main directions: similarity-based learning, data augmentation, and parameter optimization (de Andrade Porto et al., 2023). The improvements in similarity-based learning methods mainly rely on classifying by comparing the distance measures between the support set and the query set, which are generally obtained through feature extractors. Feature extractors can include commonly used networks such as ResNet (He et al., 2015), VGG (Simonyan and Zisserman, 2014), DenseNet (Huang et al., 2016), MobileNet (Howard et al., 2017), ViT (Dosovitskiy et al., 2020), Swin-TransformerV2 (Liu et al., 2021), etc. Egusquiza et al. (2022) used a ResNet network for feature extraction of disease images and then classified each extracted feature vector using a KNN classifier under the Siamese Network architecture (Chopra et al., 2005). Figueroa-Mata and Mata-Montero (2020) combined Siamese Network and CNN into a single network structure, proposing a Convolutional Siamese Network (CSN) that achieved 96% classification accuracy for crop species under few-shot conditions. Liang (2021) used DenseNet as a feature extractor and applied a Support Vector Machine (SVM) for metric classification, achieving 85.3% classification accuracy for 40 types of cotton diseases in a fewshot classification task. Nuthalapati and Tunga (2021) used Transformer to extract features and achieved an 89.4% classification accuracy for plant diseases in PlantVillage. Zhang et al. (2021) innovatively proposed the Simple Linear Image Clustering (SLIC) method, verified its performance on general datasets like Omniglot and mini-ImageNet, and demonstrated its superiority using aerial images of Pepper Plants. The data augmentation-based method primarily aims to expand the small sample data set. Scarce sample data can affect the construction of models with generalization capabilities. To address this deficiency, Riou et al. (2020) improved recognition performance on the cucumber dataset by changing the background, adjusting the lighting, and modifying the contrast of the images. Nesteruk et al. (2021) expanded the training set through basic image processing techniques such as noise addition, rotation, cropping, flipping, scaling, and image occlusion to achieve data augmentation. Another approach to image enhancement is using Generative Adversarial Networks (GANs) (Goodfellow et al., 2014) to generate large quantities of images. Khanzhina et al. (2021) used three of the most popular GAN networks to establish a pollen grain library, training and inferring according to the Siamese Network workflow. The main objective of parameter optimization-based methods is to prevent network overfitting. When the sample size per category is too small (usually less than 1000 per category), the learned parameters cannot guarantee strong generalization capabilities. Therefore, many scholars have proposed various parameter optimization techniques to ensure the learning ability of the network. Karami et al. (2020) used a modified CenterNet structure for automatic plant counting in aerial images and achieved certain performance improvements in the few-shot field. Wang et al. (2022) based on Model-Agnostic Meta-Learning (MAML) (Finn et al., 2017), determined the optimal values to use at the beginning of the training process to solve the problem of estimating vegetation density in ecological irrigation areas using aerial images.

Regarding the depth of the network, He et al. (2015) utilized a residual structure, achieving the design of a 152-layer network for the first time that could be successfully trained and inferred. The conclusions of the paper suggest that the greater the depth of the network, the higher the accuracy in image classification tasks. This also prompts us to consider whether this conclusion is universally valid for specific fewshot classification networks, and whether there is a clear metric that can quantitatively explore the impact of network depth on the performance of specific few-shot classification networks. Through extensive experiments across different datasets, we found that the last layer of features before the fully connected layer, commonly used for few-shot classification, does not enable subsequent plant disease few-shot classification tasks to achieve optimal performance. Based on this important discovery, we propose the Feature Adaptation Score (FAS) for plant disease few-shot tasks, an indicator that allows us to determine which layer of the network can perform best without testing, thus identifying outstanding network structures.

Inspired by Yang et al. (2021), we designed a plant disease feature calibration algorithm (PDFC) that can utilize features extracted from the PlantVillage dataset to calibrate features from other datasets. Unlike the method proposed by Yang, which requires simulating the distribution of meta-data and training a specialized network for classification, this paper adopts a non-parametric and training-free approach for calibration, significantly improving efficiency over the original method. Our innovations can be summarized in three points:

• We proposed a feature layer evaluation metric, Feature Adaptation Score (FAS), for plant disease few-shot tasks. Without network training, we identified the high-performance network structure Swin-Transformer V2 F6 from the backbone of Swin-Transformer V2, suitable for few-shot plant disease classification.
• Based on the new network structure mentioned above, we further proposed a few-shot plant disease feature calibration (PDFC) algorithm, suitable for the plant disease domain. This algorithm uses a small storage of feature vectors to calibrate the feature space of the target dataset, which can be combined with Swin-Transformer V2 F6 for further performance improvement.
• The effective combination of the network structure and the algorithm enables us to achieve high performance without compromising the accuracy of few-shot classification, even skipping the training phase. We validated our model and algorithm across various datasets, surpassing the performance of contemporary few-shot plant disease classification network models and achieving state-of-the-art (SOTA) results.

## 2 Materials and methods

### 2.1 Pipeline of few-shot learning

Few-shot learning (FSL) aims to classify new classes with only a few training examples per class. This capability is crucial for tasks such as plant disease classification, where obtaining large labeled datasets is challenging. In this section, we describe the pipeline for applying few-shot learning to plant disease few-shot classification. Each step is detailed below.

#### 2.1.1 Data preparation

Given dataset $D={\left\{(x_{i},y_{i})\right\}}_{i=1}^{N}$ , where *x_i_* represents an image and $y_{i}\in \{1,\ldots ,C\}$ is the corresponding label, we split the dataset into base classes $D_{base}$ and novel classes $D_{novel}$ . The base classes are used to train the feature extractor, while the novel classes are used for few-shot evaluation.

#### 2.1.2 Metric learning

Metric learning is a key component in few-shot learning. The goal of metric learning is to learn an embedding space where samples from the same class are close to each other, and samples from different classes are far apart. This is typically achieved by training a feature extractor *f_θ_* and a distance metric *d*(·,·).

#### 2.1.3 Embedding space

Given an input image *x*, the feature extractor *f_θ_* maps it to an embedding vector *z* = *f_θ_* (*x*), where $z\in {\mathbb{R}}^{d}$ and *d* is the dimensionality of the embedding space. The objective is to ensure that in this embedding space, the distance between embeddings of the same class is minimized, while the distance between embeddings of different classes is maximized.

#### 2.1.4 Distance metric

A commonly used distance metric is the L2 norm, also known as the Euclidean distance, defined as shown in Equation 1:


(1) $$d(z_{i},z_{j})={\left\|z_{i}-z_{j}\right\|}_{2}$$

where *z_i_* and *z_j_* are the embedding vectors of images *x_i_* and *x_j_*, respectively. Other distance metrics, such as cosine similarity, as shown in Equation 2: can also be used:


(2) $$d(z_{i},z_{j})=1-\frac{z_{i}\cdot z_{j}}{{\left\|z_{i}\right\|}_{2}{\left\|z_{j}\right\|}_{2}}$$

#### 2.1.5 Prototypical networks

In the context of few-shot learning, Prototypical Networks (Snell et al., 2017) are a popular approach. For each class *k*, a prototype vector *c_k_* is computed as the mean of the support set embeddings for that class as shown in Equation 3:


(3) $$c_{k}=\frac{1}{\left|S_{k}\right|}\sum_{(x_{i},y_{i})\in S_{k}}f_{\theta }(x_{i})$$

where *S_k_* is the set of support examples for class *k*. During inference, a query image *x_q_* is classified based on its distance to each class prototype, as shown in Equation 4:


(4) $${\hat{y}}_{q}=\text{arg }\underset{k}{\min\;}d(f_{\theta }(x_{q}),c_{k})$$

#### 2.1.6 Loss function

A commonly used loss in metric learning is the triplet loss. It aims to minimize the distance between an anchor *x_a_* and a positive sample *x_p_* (same class) and maximize the distance between the anchor and a negative sample *x_n_* (different class). The triplet loss is defined as shown in Equation 5:


(5) $$L=\text{max }(0,d(f_{\theta }(x_{a}),f_{\theta }(x_{p}))-d(f_{\theta }(x_{a}),f_{\theta }(x_{n}))+\alpha )$$

where *α* is a margin parameter that ensures a minimum difference between positive and negative pairs. Contrastive loss is another commonly loss used in metric learning. It operates on pairs of samples and tries to minimize the distance between similar pairs and maximize the distance between dissimilar pairs. The loss is defined as shown in Equation 6:


(6) $$L=\frac{1}{2}(y\cdot d^{2}(f_{\theta }(x_{i}),f_{\theta }(x_{j}))+(1-y)\cdot \text{max }{\left(0,m-d(f_{\theta }(x_{i}),f_{\theta }(x_{j}))\right)}^{2})$$

where y=1 if *x_i_* and *x_j_* are from the same class, y=0 otherwise, and *m* is a margin parameter.

#### 2.1.7 Training procedure

The training procedure for metric learning involves sampling batches of images and their corresponding labels, computing the embeddings using *f_θ_*, and then calculating the chosen loss function (e.g., triplet loss or contrastive loss). The model parameters *θ* are updated using gradient descent to minimize the loss, as shown in Equation 7:


(7) $$\theta \leftarrow \theta -\eta {\text{∇}}_{\theta }L$$

where *η* is the learning rate.

#### 2.1.8 Evaluation

The classic few-shot classification task follows the N-way-K-shot paradigm, where there are *N* classes, each with *K* samples. The dataset can also be represented as Equation 8


(8) $$S=\{(x_{i,j},y_{i})|i\in \{1,\ldots ,N\},j\in \{1,\ldots ,K\}\}$$

where $x_{i,j}\in {\mathbb{R}}^{H\times W\times 3}$ denotes the *j*-th sample of the *i*-th class, and $y_{i}\in \{1,\ldots ,N\}$ is the corresponding label.

For evaluation, we use standard few-shot learning metrics, such as 5-way-1-shot, 5-way-5-shot, and 5-way-10-shot classification accuracy. In an *N*-way-*K*-shot task, we randomly sample *N* classes from the novel classes and provide *K* examples per class for training. The model is then evaluated on query images from these *N* classes.

The classification accuracy *Acc* is calculated as Equation 9


(9) $$Acc=\frac{1}{\left|Q\right|}\sum_{i=1}^{\left|Q\right|}𝟙({\hat{y}}_{i}=y_{i})$$

where *Q* is the query images, ${\hat{y}}_{i}$ is the predicted label, and *y_i_* is the true label.

### 2.2 Design of STV2F6 for plant disease few-shot classification

Assume a model can be divided into *L* layers according to its depth. When an input image **x** *_i,j_* is fed into the model, the feature vector output from the *l*-th layer is denoted as *ϕ_l_* (**x** *_i,j_*), where l=1, 2,…,L .

We aim to design a versatile few-shot classification network that can effectively adapt to the characteristics of various plant disease datasets, thereby achieving efficient domain generalization capability. We ultimately chose the Swin-Transformer V2 network as our starting point for two reasons. First, this network architecture achieved the best performance on ImageNet (Krizhevsky et al., 2012) in 2022 and demonstrated remarkable capabilities across various datasets. Additionally, the network has been integrated into versions of Pytorch 1.13 and later, allowing for direct training and inference, which reduces the cost and potential for errors in reproduction. According to the design of general vision classification models, the last layer (the *L*th layer) is usually a fully connected layer. In this part, we first investigate whether the features from the penultimate layer, often used in few-shot classification, i.e., the output of the (*L* − 1)-th layer $\phi_{L-1}(x_{ij})$ , are suitable for few-shot classification in the domain of plant disease detection.

To evaluate the Feature Adaptation Score (FAS) *FAS_l_* of the feature vector $\phi_{l}(x)$ at the *l*-th layer in few-shot disease classification, we first define three parameters: the withinclass variance $\sigma_{within,l}^{2}$ , the between-class variance $\sigma_{between,l}^{2}$ , and the average between-class distance $\bar{D_{l}}$ . To derive these three parameters, we first need to calculate the mean feature vector of the samples of class *i* at the *l*-th layer, *µ_i,l_*, and the mean feature vector of all classes at the *l*-th layer, *µ_l_*. The expressions are shown in Equations 10, 11.


(10) $$\mu_{i,l}=\frac{1}{K}\sum_{j=1}^{K}\phi_{l}(x_{ij})$$

(11) $$\mu_{l}=\frac{1}{N\cdot K}\sum_{i=1}^{N}\sum_{j=1}^{K}\phi_{l}(x_{ij})$$

Based on the above discussion, we derived the expressions for the three key parameters, which are defined in Equations 12-14.


(12) $$\sigma_{within,l}^{2}=\frac{1}{N\cdot K}\sum_{i=1}^{N}\sum_{j=1}^{K}{\left\|\phi_{l}(x_{ij})-\mu_{i,l}\right\|}^{2}$$

(13) $$\sigma_{between,l}^{2}=\frac{1}{N}\sum_{i=1}^{N}{\left\|\mu_{i,l}-\mu_{l}\right\|}^{2}$$

(14) $$\bar{D_{l}}=\frac{2}{N(N-1)}\sum_{i=1}^{N}\sum_{j=i+1}^{N}\sqrt{{\left\|\mu_{i,l}-\mu_{j,l}\right\|}^{2}}$$

Each dimension of the feature vector $\phi_{l}(\text{x})$ in each layer is a linear combination of the activations of many neurons, and the value of the neurons in the *l*-th layer, before activation, is a linear combination of the activations of multiple neurons from the (*l* − 1)-th layer. Therefore, if we assume that the value of each neuron in the first layer, before the RELU activation, follows a normal distribution, then after the RELU activation, the neuron’s value follows a right-truncated normal distribution. Moreover, assuming that the number of neurons in each layer of the Swin-Transformer V2 exceeds 30, according to the Central Limit Theorem, the feature vector $\phi_{l}(\text{x})$ (before activation) in each layer follows a multivariate normal distribution. Furthermore, based on the property of linear combinations of normal distributions, and according to Equations 10, 11, $\mu_{i,l}$ and $\mu_{l}$ also follow a multivariate normal distribution, thus we have Equation 15:


(15) $$\mu_{i,l}∼N(\mu_{i},{\text{Σ}}_{i}),\;\mu_{l}∼N(\mu_{l},{\text{Σ}}_{l}).$$

According to the definition of the chi-square distribution, we can directly conclude that $\sigma_{within,l}^{2}$ and $\sigma_{between,l}^{2}$ follow a non-central chi-square distribution, which can be expressed as Equation 16:


(16) $$\sigma_{within,l}^{2}∼\chi^{2}(\lambda_{1}),\;\sigma_{between,l}^{2}∼\chi^{2}(\lambda_{2}).$$

Consider the variable $\bar{D_{l}}$ , where $\mu_{i,l}$ and $\mu_{j,l}$ are each multivariate normal variables. Specifically, $\mu_{i,l}∼N(\mu_{i},{\text{Σ}}_{i})$ and $\mu_{j,l}∼N(\mu_{j},{\text{Σ}}_{j})$ . The difference between any two feature vectors, $\mu_{i,l}$ and $\mu_{j,l}$ , is given by the difference vector $z=\mu_{i,l}-\mu_{j,l}$ . Since both $\mu_{i,l}$ and $\mu_{j,l}$ are multivariate normal, the difference vector **z** follows a multivariate normal distribution, which is shown in Equation 17:


(17) $$\text{z}∼N(\mu_{i}-\mu_{j},{\text{Σ}}_{i}+{\text{Σ}}_{j}).$$

The squared Euclidean distance between these vectors is the squared norm of **z**, given by Equation 18:


(18) $$d^{2}={\left\|\text{z}\right\|}^{2}={\text{z}}^{T}\text{z}.$$

Since **z** follows a multivariate normal distribution, the squared distance ${\left\|\text{z}\right\|}^{2}$ follows a non-central chi-square distribution, which is shown in Equation 19:


(19) $${\left\|\text{z}\right\|}^{2}∼\chi^{2}(\lambda_{3}),$$

where ${\text{λ}}_{3}$ is the non-centrality parameter, given by Equation 20:


(20) $${\text{λ}}_{3}=\frac{{\left(\mu_{i}-\mu_{j}\right)}^{T}{\left(\Sigma_{i}+\Sigma_{j}\right)}^{-1}(\mu_{i}-\mu_{j})}{2}.$$

The Euclidean distance itself is the square root of this squared distance, so the Euclidean distance between $\mu_{i,l}$ and $\mu_{j,l}$ follows the square root of a non-central chi-square distribution, as shown in Equation 21:


(21) $$\bar{D_{l}}=d=\|\text{z}\|∼\sqrt{\chi^{2}({\text{λ}}_{3})}.$$

Since the dimension directly affects the mean and variance of the non-central chi-square distribution, if a comparison is to be made, the feature vector $\phi_{l}(\text{x})$ in each layer needs to be scaled by its dimension so that the feature vectors of all layers are compared on the same scale. After performing the dimensional scaling, we can define *FAS_l_*,


(22) $$FAS_{l}=\frac{\sigma_{between,l}^{2}/n}{\sigma_{within,l}^{2}/n}+\frac{\bar{D_{l}}}{\sqrt{n}}=\frac{\sigma_{between,l}^{2}}{\sigma_{within,l}^{2}}+\frac{\bar{D_{l}}}{\sqrt{n}}.$$

We will explain why *FAS_l_* takes the form of Equation 22. First, we discuss why the scaling factors of the three parameters are not exactly the same. The first two variables, $\sigma_{between,l}^{2}/n$ and $\sigma_{within,l}^{2}/n$ , represent the variance of the means. According to the law of large numbers, the variance of the sample mean is the original variance divided by the sample size *n*, meaning the variance of the mean decreases as the sample size *n* increases. This is why the first two variables use 1/n ; they reflect the influence of sample size on the variance of the mean. The last variable, $\bar{D_{l}}/\sqrt{n}$ , represents the average Euclidean distance between sample pairs, which involves averaging the distances between multiple sample pairs. Since the standard error of the mean is the sample standard deviation *σ* divided by $\sqrt{n}$ , we use $1/\sqrt{n}$ for the last term to account for the influence of the standard error. As the sample size *n* increases, the estimation of the mean becomes more precise, which is why we adjust the variance using $1/\sqrt{n}$ . In summary, the first two terms use 1/n , while the third term uses $1/\sqrt{n}$ , allowing the three different variables to have values on the same scale, thereby making the calculation of *FAS_l_* more stable.

Another aspect we need to further clarify is why the two terms in *FAS_l_* are combined using addition rather than multiplication. This is primarily because these two terms measure different statistics and do not have a direct dependency or interaction. The first term, $\sigma_{between,l}^{2}/\sigma_{within,l}^{2}$ , reflects the relative dispersion between and within the feature distributions, measuring the ratio of differences between categories to differences within the same category, describing the spread of the data. The second term, $\bar{D_{l}}/\sqrt{n}$ , reflects the precision of the sample mean, specifically the average Euclidean distance between sample pairs divided by $\sqrt{n}$ , meaning that as the sample size increases, the estimation of the mean becomes more precise. Since these two measures independently reflect different aspects of the data, there is no direct interaction between them, and addition is used to accumulate their effects instead of multiplication. Addition is more natural and appropriate because it effectively combines these two measures into a single composite score, while multiplication is typically used when there is an interaction or amplifying effect between the measures. Therefore, addition is the choice that aligns better with statistical intuition.

The ideal feature vector $\phi_{l}(x)$ for calculating the distance to the prototype should have low within-class variance, high between-class variance, and a large between-class distance. This ensures that the model has good consistency for samples within the same class while maintaining high discriminability for samples of different classes. The more suitable the feature vector $\phi_{l}(x)$ is for the Plant Disease Few-Shot task, the higher the corresponding *FAS_l_* should be. Equation 15 meets this requirement. We refer to the new network structure discovered through *FAS_l_* as Swin-Transformer V2 F6 (STV2F6).

### 2.3 Plant disease feature calibration

**Figure 1:**
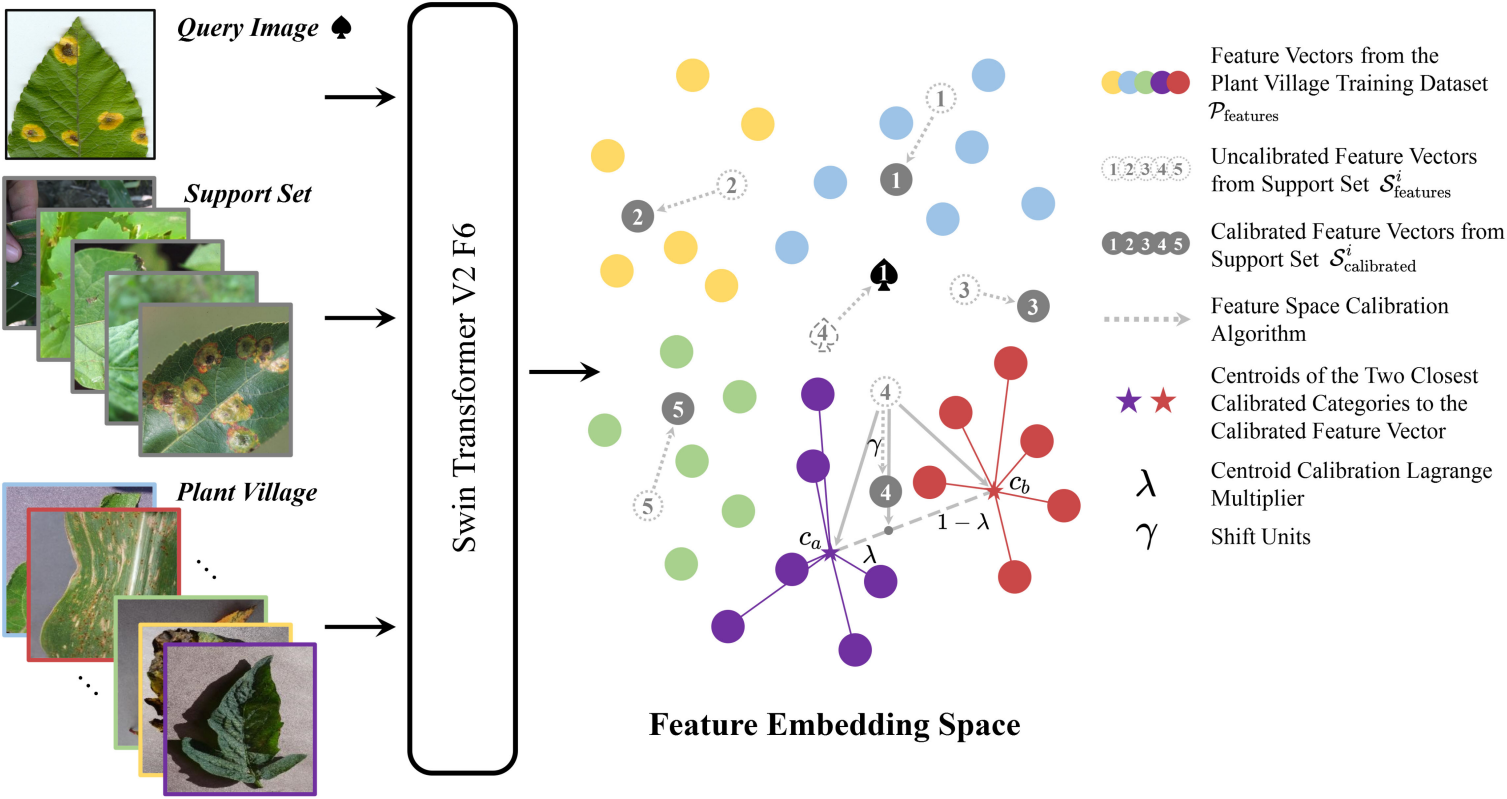
Schematic diagram of plant disease feature calibration algorithm.

Plant disease few-shot classification inherently has sparse training samples. Therefore, if existing data can assist in the N-way-K-shot task, it can further improve the classification accuracy of this task. Inspired by the article (Yang et al., 2021), we attempted to use some data from PlantVillage to calibrate the feature vectors of the support set and query set in the N-way-Kshot task. However, unlike the method mentioned in (Yang et al., 2021), we did not train an additional classification network to learn the distribution of the feature space. Instead, we used an intuitive and convenient non-parametric calibration method. The algorithm framework is shown in **Figure 1**.

From the figure, it can be seen that the query set Q and the support set S both undergo Swin-Transformer V2 F6 to obtain feature vectors $Q_{\text{features}}$ and $S_{\text{features}}$ , respectively. The entire PlantVillage training set P also undergoes Swin-Transformer V2 F6 to obtain the feature vector set $P_{\text{features}}$ , which serves as a reference for calibrating the feature vectors $S_{\text{features}}$ _s_. The calibration steps are as follows: For each 5-way-1-shot task, each query image q∈Q corresponds to a feature vector $q_{\text{features}}\in Q_{\text{features}}$ . For category *i*, the feature vector set $P_{\text{features}}$ of PlantVillage calculates the L2 distance *d^i^* to the support set $S_{\text{features}}^{i}$ of that category. These distances are sorted in ascending order as $d_{1}^{i}\leq d_{2}^{i}\leq \ldots \leq d_{\left|P\right|}^{i}$ , and the top m smallest distances $d_{1}^{i}\leq d_{2}^{i}\leq \ldots \leq d_{m}^{i}$ are selected. The centroid of these m vectors is calculated as $c_{i}=\frac{1}{m}\sum_{k=1}^{m}d_{k}^{i}$ . We use the centroids of all categories obtained by this method to calibrate $S_{\text{features}}^{i}$ . For category *i*, we first calculate the L2 distances between the category centroid $c^{i}$ and $S_{\text{feature}}^{i}$ and sort them in ascending order as ${\hat{d}}_{1}^{i}\leq {\hat{d}}_{2}^{i}\leq \ldots \leq {\hat{d}}_{5}^{i}$ . The two smallest distances ${\hat{d}}_{1}^{i}$ and ${\hat{d}}_{2}^{i}$ correspond to centroids $c_{a}$ and $c_{b}$ . A point T is selected on the line connecting these two centroids, with the position of T determined by the hyperparameter λ . The $S_{\text{features}}^{i}$ is then shifted γ units in the direction collinear with T, completing the calibration of the support set $S_{\text{features}}^{i}$ to obtain the calibrated support set $S_{\text{calibrated}}^{i}$ . The above steps are repeated to calibrate all support sets. Finally, the distances $d_{qS^{i}}^{i}$ between all calibrated support sets $S_{\text{calibrated}}^{i}$ and the query feature vector $q_{\text{features}}$ are calculated, and the label for the query set *q* is obtained as $q_{\text{labels}}=\arg{\text{min}}_{i}d_{qS^{i}}^{i}$ . For the 5-way-5-shot and 5-way-10-shot tasks, it is sufficient to use the average of the support set features $S_{\text{feature}}^{i}$ . The implementation process of the algorithm is presented in pseudocode form in **Algorithm 1**.

Algorithm 1 **PDFC Algorithm.**

**Input**: Query set 𝒬, Support set 𝒮, PlantVillage training set
 
P
, Lagrange multiplier *λ*, Distance shift units *γ*
**Output**: Query set labels 
$q_{\text{labels}}$

1: Calculate feature vectors 
$Q_{\text{features}}$
, 
$S_{\text{features}}$
, and 
$P_{\text{features}}$
 using Swin-Transformer V2 F6
2: **for** each query image 
q∈Q
 **do**
3:              *q*
_features_ ← STV2F6(*q*)
4:              **for** each category *i* **do**
5:              Calculate L2 distance *d^i^
* between 
$P_{\text{features}}$
 and 
$S_{\text{features}}^{i}$

6:              Sort *d^i^
* in ascending order: 
$d_{1}^{i}\leq d_{2}^{i}\leq \ldots \leq d_{\left|P\right|}^{i}$

7:              Select the top *m* distances: 
$d_{1}^{i},d_{2}^{i},\ldots ,d_{m}^{i}$

8:              Calculate centroid: 
$c_{i}=\frac{1}{m}\sum_{k=1}^{m}P_{\text{features}}^{i,m}$

9:              **end for**
10:              **for** each category *i* **do**
11:              Calculate L2 distances 
${\hat{d}}^{i}$
 between *c^i^
* and 
$S_{\text{features}}^{i}$

12:              Sort 
${\hat{d}}^{i}$
 in ascending order: 
${\hat{d}}_{1}^{i}\leq {\hat{d}}_{2}^{i}\leq \ldots \leq {\hat{d}}_{5}^{i}$

13: Select the top 2 smallest distances: 
${\hat{d}}_{1}^{i},{\hat{d}}_{2}^{i}$
 corresponding to centroids 
$c_{a},\;c_{b}$

14: Determine point T on the line connecting *c_a_
* and *c_b_
* using Lagrange multiplier *λ*
15: Shift 
$S_{\text{features}}^{i}$
 *γ* units toward T to obtain 
$S_{\text{calibrated}}^{i}$

16:              **end for**
17: **end for**
18: **for** each query image 
q∈Q
 **do**
19: Calculate distances 
$d_{qS^{i}}^{i}$
 between 
$q_{\text{features}}$
 and 
$S_{\text{calibrated}}^{i}$

20:              
$q_{\text{labels}}\leftarrow \arg\min_{i}d_{qS^{i}}^{i}$

21: **end for**
22: **Return:** 
$q_{\text{labels}}$

## 3 Results

### 3.1 Environment platform

The hardware setup for the entire experiment is as follows: Processor: Intel(R) Core(TM) i7–10700 CPU @ 2.90GHz, 16GB RAM, NVIDIA GeForce RTX 3060 12GB GPU. Software environment: Python 3.9, Pytorch 1.13.1.

### 3.2 Implementation details

We first train Swin-Transformer V2-T on the ImageNet dataset using the stochastic gradient descent (SGD) optimizer (momentum of 0.9 and weight decay of 4 × 10^−5^). The initial learning rate is set to 0.05, and adjusted using step decay with a decay factor of 0.1 every 30 epochs. The model is trained for 300 epochs with a batch size of 128. Data augmentation includes random cropping, horizontal flipping, and color jittering, and the input image size is resized to 224×224. Dropout is set to 0.2, and L2 regularization is used to further enhance the model’s generalization ability. Under these settings, the feature extractor achieves a Top-1 accuracy of 73.18% on the ImageNet dataset. After pre-training, the last layer block, F7, is removed to obtain the feature extractor STV2F6, which is then used for comparative experiments on various datasets with STV2F6+PDFC.

Before the comparative experiments, due to the small number and narrow range of hyperparameters in the PDFC algorithm, we first use Bayesian optimization to find the best combination as the nearest hyperparameter combination for the current dataset, and then perform the final large-scale experiments. Finally, to ensure the stability of the experimental results, each set of N-way-K-shot data in the experiments is sampled 1,000 times, and the average of 20 experimental results is obtained after cross-validation.

### 3.3 Datasets

*CUB* (Wah et al., 2011) dataset contains 11,788 images of 200 bird species, and is a classic dataset in the field of fine-grained object classification. Each image is annotated with detailed information, such as the bird species, morphological features, and colors. It is widely used in bird classification and object detection research, especially in visual recognition applications.

*mini-ImageNet* (Vinyals et al., 2016) is a subset of ImageNet, designed to provide a standard evaluation platform for few-shot learning. It contains 100 categories, with 600 images per category, covering various everyday objects. mini-ImageNet is widely used in testing and research for few-shot learning algorithms, particularly in convolutional neural networks (CNNs) and meta-learning methods.

*PlantVillage* (Hughes and Salathé, 2015) is an epidemiological dataset used for evaluating automated plant disease recognition systems. All images were collected in a laboratory setting and include images of both healthy and diseased plant leaves. Additionally, the dataset includes augmented images obtained through operations such as flipping, gamma correction, noise injection, PCA color augmentation, rotation, and scaling, encompassing a total of 38 plant diseases and 61,486 images, making it one of the most crucial evaluation datasets in the field of plant disease research.

*PlantDoc* (Singh et al., 2019) is an open-source dataset for plant disease diagnosis, containing over 4,000 images, covering 13 crop species and 26 types of plant diseases. This dataset is particularly suitable for few-shot learning and transfer learning tasks, providing researchers with a rich resource for training and evaluating plant disease classification models.

*Plant Real-World (* Li et al., 2023) is a small-scale crop disease diagnosis dataset, containing samples of multiple diseases from four common crops: rice, wheat, maize, and soybean, with a total sample size exceeding 1,000. The dataset includes 12 types of diseases and all samples are captured from field images and disease maps. With a complex and diverse background, this dataset can be used for researching the cross-domain generalization issues in few-shot learning.

*Plant&Pest* (Li and Yang, 2021) is another small dataset used to validate the performance of few-shot classification models, which includes two parts: plants and pests. In this experiment, we used the plant part of the dataset for few-shot classification experiments.

**Figure 2:**
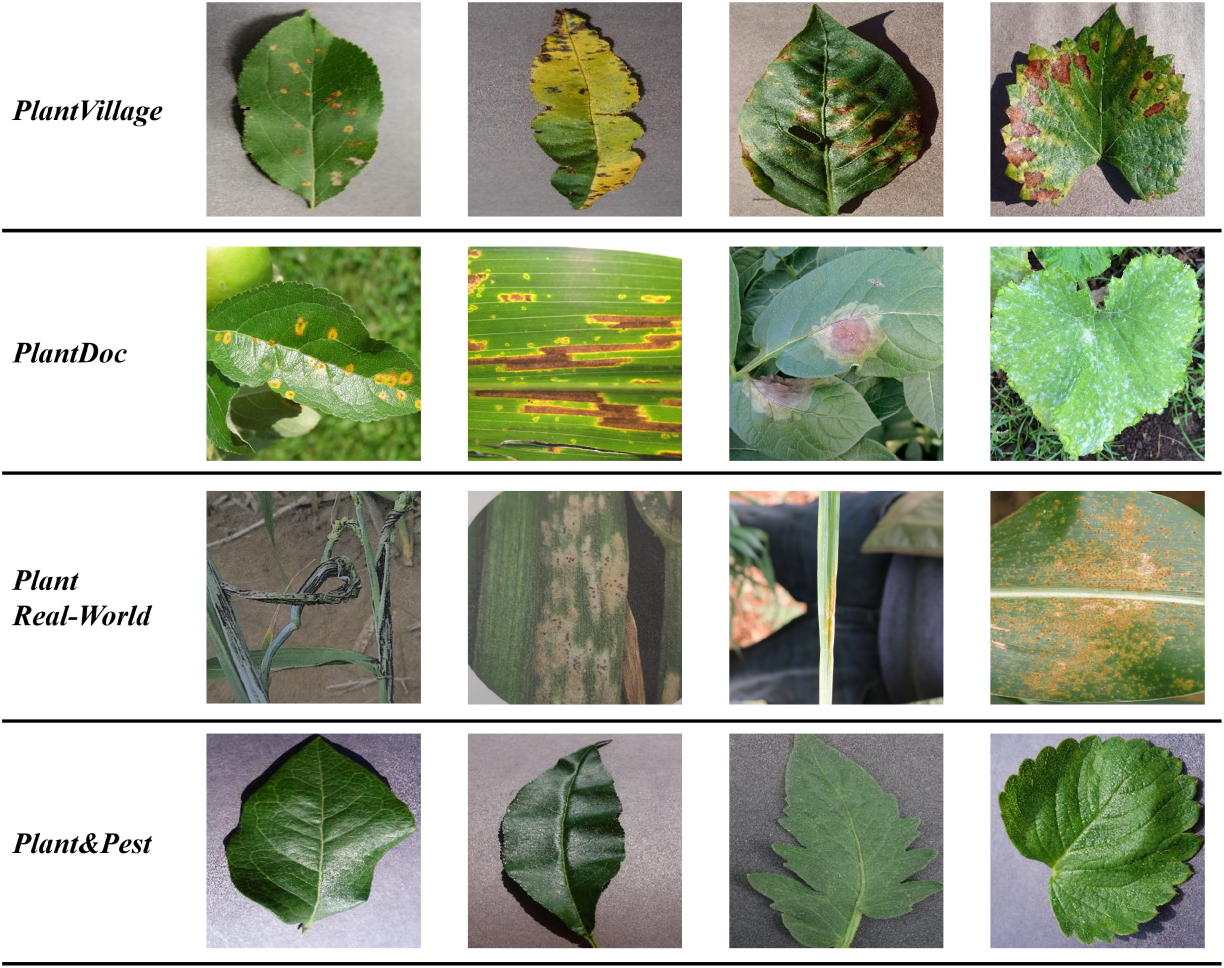
Examples of images from four crop datasets.

The illustrations of the four crop datasets used in the experiment are listed in **Figure 2**.

### 3.4 Results

We use the lightweight version of Swin-Transformer V2, Swin-Transformer V2-T, and employ its pre-trained weights on ImageNet-1K (Krizhevsky et al., 2012) to extract features for the query set Q. According to the structure in the original paper, SwinTransformer V2-T consists of a total of 7 large blocks, each of which outputs a feature *ϕ_l_* (**x**) as the input to subsequent components. The output of the 7th block, *ϕ*_7_(**x**), is directly connected to the fully connected layer. To verify whether the final feature *ϕ*_7_(**x**), similar to conventional few-shot tasks, is also suitable for the Plant Disease Few-Shot task, we input all images from six datasets into the backbone of SwinTransformer V2-T. This allows us to obtain 7 sets of features *ϕ*_1∼7_(**x**) generated in the backbone for the six datasets, as well as the corresponding Feature Adaptation Scores *FAS*_1∼7_, as shown in **Table 1**.

**Table 1:** Performance adaptation score table *FAS*_1∼7_.

Dataset	*FAS*_1_	*FAS*_2_	*FAS*_3_	*FAS*_4_	*FAS*_5_	*FAS*_6_	*FAS*_7_
CUB	4.57	6.58	6.93	13.34	18.63	**24.71**	19.11
mini-ImageNet	8.10	9.36	13.37	17.84	17.48	**20.30**	18.09
PlantVillage	6.68	11.63	10.21	18.21	17.83	**29.92**	11.40
PlantDoc	2.92	4.13	3.66	5.70	6.18	**9.26**	4.83
Plant Real-World	7.95	12.61	10.75	20.06	19.43	**30.48**	9.86
Plant&Pest	7.29	13.31	12.27	22.38	21.83	**37.25**	11.85

*FAS*_1∼7_ units are all ×10^−2^.

Bold values highlight the best results obtained by our method in comparison with existing approaches.

**Figure 3:**
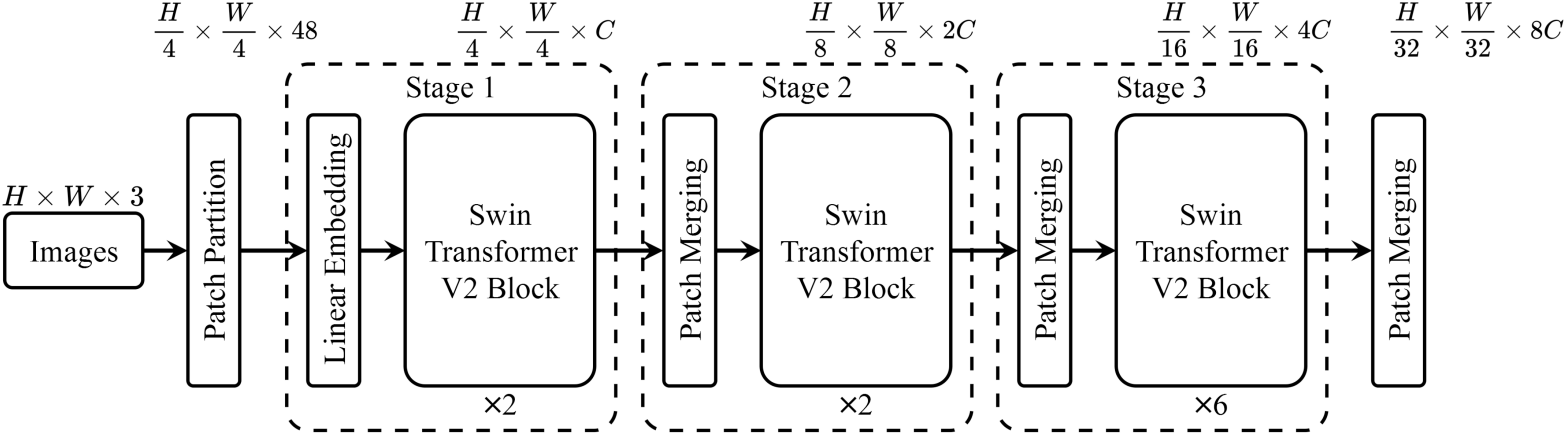
The architecture of Swin-Transformer V2-F6.

As shown in **Table 1**, the score *FAS*_6_ corresponding to the sixth layer feature (Feature Six, F6) of Swin-Transformer V2-T far exceeds the scores of all other layers, indicating that the sixth layer feature F6 is more suitable for the Plant Disease Few-Shot task than the features from other layers. We then conducted comparative experiments using the sixth layer feature F6 and the most commonly used seventh layer feature F7 on different datasets to examine the accuracy of the Plant Disease Few-Shot task. The results are shown in **Table 2**. From the experimental results, it is evident that F6, with the highest score, achieved a significant improvement in accuracy for the Plant Disease Few-Shot task compared to the commonly used F7. Therefore, both the Performance Adaptation Score *PAS* and the accuracy comparison of the Plant Disease Few-Shot task’s test set confirm that F6 is more suitable for the Plant Disease Few-Shot task than F7. We consider this an important finding in the field of plant disease diagnosis. Based on the above experimental results, we propose that the most suitable feature extraction network structure for the Plant Disease FewShot task is Swin-Transformer V2 F6 (STV2F6), as shown in **Figure 3**.

**Table 2:** Comparison of few shot task performance between F6 and F7 on different datasets.

Dataset	5W1S	5W5S	5W10S
CUB[F7]	62.39	71.81	74.29
CUB[F6]	91.96	94.14	96.14
mini-ImageNet[F7]	64.25	77.52	80.71
mini-ImageNet[F6]	76.41	88.26	90.03
PlantVillage[F7]	40.95	51.42	53.38
PlantVillage[F6]	68.78	90.89	94.04
PlantDoc[F7]	28.57	36.25	37.02
PlantDoc[F6]	36.84	55.38	62.00
Plant Real-World[F7]	48.50	72.97	78.54
Plant Real-World[F6]	69.44	84.55	87.61
Plant&Pest[F7]	42.57	53.03	55.28
Plant&Pest[F6]	75.80	95.35	97.40

Further, we use STV2F6 as a fixed feature extractor and apply the PDFC algorithm to calibrate the feature space of the features extracted from this structure. At the same time, we compare the performance results of STV2F6 combined with the PDFC algorithm with various state-of-the-art methods in the field of few-shot learning. The evaluation metrics for the comparison are the standard few-shot learning metrics: 5-way-1-shot, 5-way-5-shot, and 5-way-10-shot classification accuracy. We first compare our method with several recent few-shot works on the two general datasets, CUB and miniImageNet. During the experiments, the feature vectors in the database are all computed from the samples in ImageNet. The results are shown in **Table 3**. It is evident from **Table 3** that STV2F6+PDFC significantly outperforms the latest research results due to the calibration of the target domain using feature vectors from the ImageNet training set.

**Table 3:** Comparison results of few-shot task on cub and mini-ImageNet.

Model	CUB		mini-ImageNet	
	5WIS	5W5S	5WIS	5W5S
RestoreNet (Xue and Wang, 2020)	74.32	–	59.28	–
RAP-ProtoNet (Hong et al., 2021)	75.17	88.29	53.64	74.54
MAML (Finn et al., 2017)	55.92	72.09	58.37	69.76
MultiSem (Schwartz et al., 2019)	76.1	82.9	67.3	82.1
CPDE (Zou et al., 2020)	80.11	89.28	63.21	79.68
CFA (Hu et al., 2019)	73.90	86.8	63.21	79.68
Neg-Cosine (Liu et al., 2020)	72.66	89.40	63.85	81.57
CentAlign (Afrasiyabi et al., 2019)	74.22	88.65	59.88	80.35
DC (Hu et al., 2019)	77.22	89.58	66.91	80.74
SRestoreNet (Xue and Wang, 2020)	76.85	–	61.14	–
EPNet (Rodr’iguez et al., 2020)	82.85	91.32	66.50	81.06
ICI (Wang et al., 2020)	87.87	92.38	65.77	78.94
TIM-GD (Boudiaf et al., 2020)	82.2	90.8	73.9	85.0
LaplacianShot (Ziko et al., 2020)	80.96	88.68	72.11	82.31
RAP-LaplacianShot (Hong et al., 2021)	83.59	90.77	74.29	84.51
BD-CSPN (Liu et al., 2019)	84.90	90.22	65.94	79.23
STV2F6+PDFC	**91.26**	**94.14**	**76.41**	**88.26**

Bold values highlight the best results obtained by our method in comparison with existing approaches.

In addition, we compared our approach with various methods recently used in crop disease few-shot tasks, and the comparison results across four datasets are shown in **Tables 4**-**7**. From the results, it is clear that the STV2F6+PDFC structure outperforms recent methods in terms of classification accuracy, recall, and F1-score for crop disease few-shot tasks. The experimental results across multiple datasets also clearly demonstrate that the FAS metric can accurately identify the best network structure for fewshot tasks. Meanwhile, the PDFC algorithm can adjust the target domain’s sample feature vectors toward the source domain (ImageNet or PlantVillage), enabling STV2F6+PDFC to efficiently and accurately classify test samples, even when there are no large numbers of reference samples during the testing phase, by leveraging the features learned from previous training.

**Table 4:** Comparison results of plant disease few-shot task on PlantVillage.

Model	5W is			5W5S			5W10S		
	Precision	Recall	F1	Precision	Recall	F1	Precision	Recall	F1
Li and Yang (2021)	81.10	80.45	80.77	87.00	82.32	84.64	90.40	85.12	87.74
Yang et al. (2022)	85.38	83.21	84.29	88.12	85.57	86.83	90.59	86.14	88.32
Huang et al. (2022)	83.32	81.56	82.43	89.12	84.73	86.83	91.67	88.28	89.96
Zhang et al. (2021)	80.56	78.89	79.71	85.82	80.68	83.13	88.32	82.54	85.35
Rezaei et al. (2024)	86.23	85.67	85.95	92.01	88.12	90.01	94.23	90.11	92.06
STV2F6+PDFC	**91.81**	**91.39**	**91.60**	**95.32**	**93.26**	**94.27**	**95.50**	**94.12**	**94.81**

The unit of F1 Score in the table is 10^−2^.

Bold values highlight the best results obtained by our method in comparison with existing approaches.

**Table 5:** Comparison results of plant disease few-shot task on PlantDoc.

Model	5W1S			5W5S			5W10S		
	Precision	Recall	F1	Precision	Recall	F1	Precision	Recall	F1
Li and Yang (2021)	37.58	50.43	43.12	51.24	58.99	54.83	58.96	64.53	61.61
Yang et al. (2022)	37.95	52.36	43.96	52.77	60.42	56.33	55.63	68.12	61.20
Huang et al. (2022)	37.69	50.76	43.22	51.25	55.71	53.38	56.34	68.23	61.70
Zhang et al. (2021)	38.63	51.34	44.06	50.41	57.39	53.65	57.32	64.90	60.86
Rezaei et al. (2024)	44.27	55.12	49.07	62.49	62.01	62.25	70.74	70.63	70.69
STV2F6+PDFC	**49.29**	**56.29**	**52.54**	**65.88**	**63.43**	**64.63**	**72.00**	**70.12**	**71.05**

Bold values highlight the best results obtained by our method in comparison with existing approaches.

**Table 6:** Comparison results of plant disease few-shot task on plant real-world.

Model	5W1S			5W5S			5W10S		
	Precision	Recall	F1	Precision	Recall	F1	Precision	Recall	F1
Li and Yang (2021)	54.18	55.23	54.69	67.39	70.12	68.71	70.64	75.89	73.16
Yang et al. (2022)	55.31	57.45	56.35	66.41	68.34	67.36	71.49	78.23	74.72
Huang et al. (2022)	57.06	60.12	58.53	70.91	75.67	73.22	72.43	80.45	76.23
Zhang et al. (2021)	58.21	59.87	59.02	66.33	69.89	68.05	69.86	77.12	73.32
Rezaei et al. (2024)	64.88	66.34	65.60	80.23	82.01	81.11	84.25	88.76	86.43
STV2F6+PDFC	**70.95**	**68.41**	**69.64**	**85.62**	**82.57**	**84.06**	**88.12**	**89.98**	**89.04**

Bold values highlight the best results obtained by our method in comparison with existing approaches.

**Table 7:** Comparison results of plant disease few-shot task on plant & pest.

Model	5W1S			5W5S			5W10S		
	Precision	Recall	F1	Precision	Recall	F1	Precision	Recall	F1
Li and Yang (2021)	84.58	85.23	84.90	89.34	90.12	89.73	92.78	93.89	93.33
Yang et al. (2022)	81.63	83.45	82.53	87.45	88.34	87.89	91.81	92.23	92.02
Huang et al. (2022)	85.72	86.12	85.92	90.63	91.67	91.15	93.71	94.45	94.08
Zhang et al. (2021)	84.96	85.87	85.41	89.74	90.89	90.31	93.65	94.12	93.88
Rezaei et al. (2024)	93.21	94.34	93.77	95.36	96.01	95.68	95.93	96.76	96.34
STV2F6+PDFC	**97.09**	**96.41**	**96.75**	**98.42**	**97.57**	**97.99**	**98.46**	**98.98**	**98.72**

Bold values highlight the best results obtained by our method in comparison with existing approaches.

**Figure 4:**
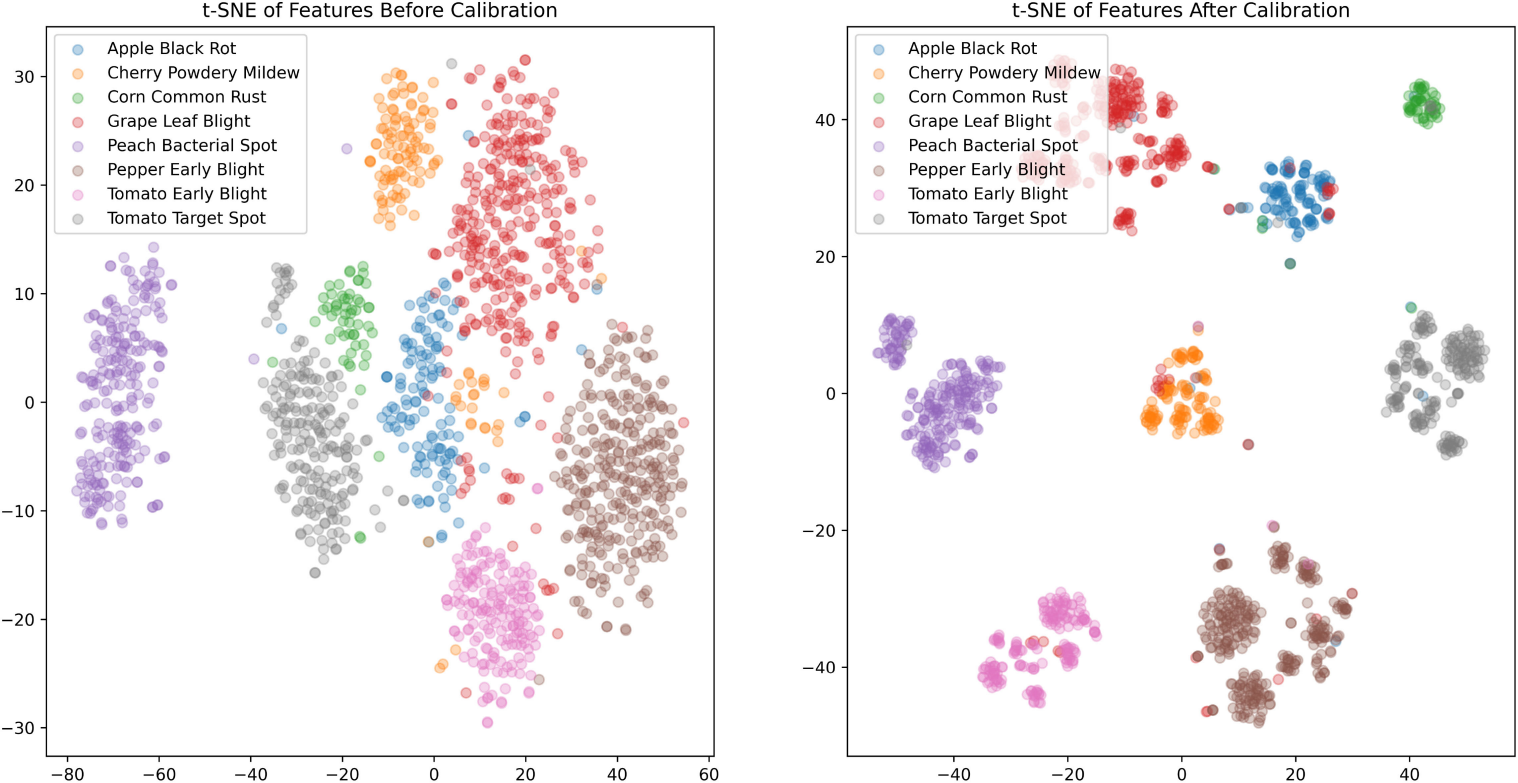
Feature distribution visualization before and after using the PDFC algorithm in STV2F6.

To further explore the direct impact of the PDFC algorithm on calibrating the embedding feature space, we visualized the feature distributions of STV2F6 before and after calibration using the t-SNE (van der Maaten and Hinton, 2008) tool. The visualization results on the PlantVillage test set are shown in **Figure 4**. As can be seen from the figure, the features calibrated using the PDFC algorithm are more compactly clustered within the same class, and different classes are further apart, with less overlapping distribution areas. This indicates that it is an effective feature calibration method.

### 3.5 Sensitivity analysis of the experimental parameters

The parameters affecting the accuracy of the STV2F6+PDFC algorithm experiment results are numerous. In terms of training paradigms, the main aspects include whether to use a pre-trained model, whether to perform finetuning. The parameters mainly involve two hyperparameters, namely the Lagrange Multiplier *λ* and the Shift Units *γ*. Below, we discuss and experiment with the stability of the algorithm in terms of training paradigms and parameters.

In our previous experimental results, we directly used the pre-trained model of Swin-Transformer V2-T without any finetuning. We will now conduct separate experiments on whether or not to use a pre-trained model, whether or not to finetune, and whether or not to use the PDFC algorithm. Since the STV2F6 model must either be pre-trained or trained from scratch, a model that neither requires pre-training nor finetuning does not exist. Therefore, there are a total of six remaining scenarios. We conducted separate experiments for these six scenarios on PlantVillage, and the experimental results are shown in **Table 8**. In the table, the fine-tuning option refers to training the STV2F6 from scratch if no pre-trained model is used. As shown in the table, the model using the pre-trained model and fine-tuning, combined with the PDFC algorithm, exhibits a significant performance drop compared to the model without fine-tuning. This can be explained by the catastrophic forgetting phenomenon that occurs after fine-tuning the neural network. Since few-shot tasks impose higher requirements on the model’s generalization ability, fine-tuning focused on the dataset disrupts some of the already optimized weights of the pre-trained model. Additionally, the results of this experiment show that by directly using the pre-trained model with the PDFC algorithm, without any parameter training, not only does it skip the model training step, but it also achieves the best 5W1S task accuracy. This is an important first attempt in the field of plant disease classification, emphasizing to some extent that the model’s generalization ability should be the primary consideration in this field. Fine-tuning and further algorithm optimization should only be considered for models with poor generalization ability.

**Table 8:** Comparison results of plant disease few-shot task on PlantVillage.

Model	Pretrained	Fine-tune	PDFC	5W1S
STV2F6		✓		61.09
		✓	✓	61.25
	✓			68.78
	✓		✓	**91.81**
	✓	✓		75.12
	✓	✓	✓	76.57

Bold values highlight the best results obtained by our method in comparison with existing approaches.

“√” denotes the use of the corresponding method.

**Figure 5:**
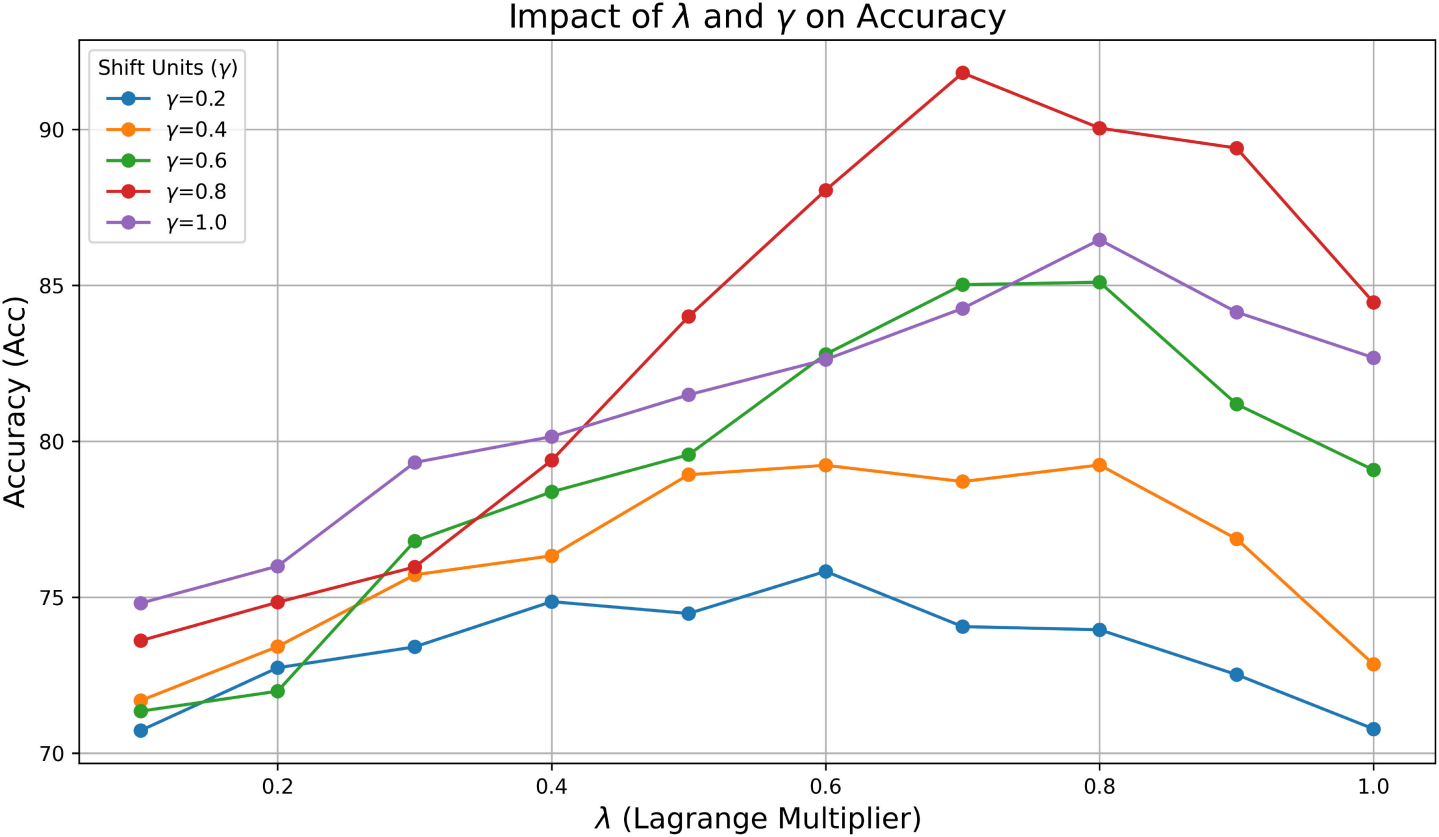
The impact of hyperparameters *λ* and *γ* on the performance of the PDFC algorithm.

In addition to training paradigms, the parameters mainly involve two hyperparameters, the Lagrange Multiplier *λ* and the Shift Units *γ*, which affect the enhancement effect of the PDFC algorithm on the model. We used grid search to test different Lagrange Multiplier *λ* and Shift Units *γ* on PlantVillage. The impact of different *λ* and *γ* on the performance of STV2F6 is shown in **Figure 5**. We searched *λ* and *γ* with step sizes of 0.1 and 0.2, respectively. The different parameter combinations have a crucial impact on the model’s performance. Since these two parameters have definite meanings mathematically, we can define their ranges as [0,1]. Thanks to the small number of parameters and the lack of need for training, even with grid search, all results can be obtained in a very short time.

In our experiments, we need to select different values for the Lagrange Multiplier *λ* and Shift Units *γ* based on the dataset. When dealing with large datasets, we can use Bayesian optimization to efficiently search for the best combination of *λ* and *γ*. First, we define the parameter space for *λ* ∈ [10^−3^,10^1^] and for *γ* ∈ [10^−3^,10^1^]. Bayesian optimization uses a Gaussian model to simulate the performance of different hyperparameter combinations. It then employs the Expected Improvement acquisition function to balance exploration and exploitation, guiding the search for the optimal hyperparameters. On the largest datasets, we evaluate through 100 iterations, with the algorithm selecting the best-performing combination of *λ* and *γ* based on the model’s performance on the validation set. This process allows for efficient hyperparameter search, especially in large-scale experiments where computational resources are limited. Finally, after the optimization process, the best combination of *λ* and *γ* is used for the final largescale experiments, ensuring the model’s optimal performance on the target dataset.

## 4 Discussion

In exploring the STV2F6 structure, we also tested whether the FAS parameter could identify the optimal depth range of various network structures on backbone networks of mainstream architectures, including ResNet, VGG, MobileNet, and ShuffleNet. The actual test results show that the size of FAS is still strictly positively correlated with the PDFS test accuracy using the pre-trained model directly. However, due to the insufficient accuracy of the above networks themselves and issues such as the inability to perform feature calibration with the PDFC algorithm or the lack of significant calibration effects, they cannot be compared on the same level with the latest models using pre-training, fine-tuning, and network structure adjustments. Therefore, we used Swin-Transformer V2-T as the backbone network to maximize the structural finding ability of FAS. It can be considered that the STV2F6 structure is a successful practice guided by theory.

As shown in the experimental results in **Table 4**, the performance of STV2F6+PDFC on the 5W10S task in PlantVillage has already approached the accuracy of the standard model (Afifi et al., 2020) trained with full supervision. On one hand, this demonstrates the excellent performance of our algorithm. On the other hand, it also raises the question of whether we need a more complex standard dataset to replace PlantVillage. It is well known that PlantVillage holds a similar position in plant disease recognition as ImageNet does in image classification. However, due to the rapid evolution of neural network structures, STV2F6 can achieve remarkable classification accuracy by using the pre-trained model directly without fine-tuning, even with only 10 images per class. Therefore, it might be more beneficial for the development of smart agriculture to supplement the PlantVillage benchmark, for example, by using PlantDoc as the benchmark instead of PlantVillage. This could encourage future researchers to focus more on datasets with more complex backgrounds, such as PlantDoc, thereby enhancing the practicality of the algorithms.

Seeing the performance results of STV2F6+PDFC, we also envision future work. In terms of the algorithm, we plan to apply the PDFC algorithm to a variety of frameworks, rather than being deeply tied to STV2F6. As can be seen from the implementation process of the PDFC algorithm, it does not depend on the configuration of the feature extraction network. With the advent of GPT-4 (Achiam et al., 2023), we are preparing to replace STV2F6 with a Transformer-based large language model framework to explore the potential of the model in the field of natural language processing. In terms of improving model performance and efficiency, we intend to further refine the feature vector database of the PDFC algorithm by using representative sample feature vectors for comparison, rather than using the feature vectors of the entire training set for retrieval. This approach can significantly reduce the forward inference and feature vector calibration time, especially when the dataset is large.

## 5 Conclusion

Through in-depth research on few-shot classification tasks for plant disease identification, this paper identifies a highp-erformance network structure, Swin-Transformer V2 F6, using the Feature Adaptation Score (FAS) metric without network training and fine-tuning. Based on this structure, we propose a Plant Disease Feature Calibration (PDFC) algorithm that complements it. Extensive experiments on different datasets show that the Swin-Transformer V2 F6 network structure, evaluated and selected using FAS, combined with the PDFC algorithm, significantly improves the accuracy of few-shot plant disease classification, surpassing existing models and achieving state-of-the-art performance. Our research provides an efficient and accurate method for addressing plant disease classification with few-shot data, offering both theoretical innovation and practical value. It provides robust support for the automatic identification of rare plant diseases and agricultural production management.

## Data Availability

The original contributions presented in the study are included in the article/supplementary material. Further inquiries can be directed to the corresponding author.
